# Study of Mechanical Response of Paper-Based Microfluidic System as a Potential Milk Tester

**DOI:** 10.3390/mi14071380

**Published:** 2023-07-06

**Authors:** Laura Alejandra Ireta-Muñoz, Isaías Cueva-Pérez, David Alejandro Elvira-Ortiz, Leonardo Esteban Moreno-Suárez, Ángel Pérez-Cruz

**Affiliations:** Facultad de Ingeniería, Universidad Autónoma de Querétaro, Cerro de las Campanas s/n Querétaro, Querétaro 76000, Mexico; lireta08@alumnos.uaq.mx (L.A.I.-M.); isaias.cueva@uaq.mx (I.C.-P.); david.elvira@uaq.mx (D.A.E.-O.); leonardo.moreno@uaq.mx (L.E.M.-S.)

**Keywords:** microfluidic system, electro-mechanical response, hygroexpansion, cantilever beam paper-based, deflection

## Abstract

Milk is considered a complete meal that requires supervision to determine its suitability for human consumption. The development of sustainable devices that evaluate food properties has gained importance due to the necessity of integrating these instruments into the production chain. However, the materials employed to develop it, such as polymers, semiconductors, and glass, lack sustainability and require specialized equipment to fabricate them. Different chemical techniques have been used to miniaturize these detection systems such as microfluidics, which have been used in milk component detection using colorimetry. In this work, a cantilever beam paper-based microfluidic system is proposed to evaluate differences in milk, according to nutritional information, using its electromechanical response. A 20-microliter milk drop is deposited in the system, which induces hygroexpansion and deflection due to liquid transport within the paper. Likewise, a conductive path is added on the beam top surface to supply a constant current that induces heat to evaporate the solution. According to the results obtained, it is possible to point out differences between trademarks with this microfluidic system. The novelty of this system relies on the paper electromechanical response that integrates the hygroexpansion-induced displacement, which can be used for further applications such as milk microtesters instead of colorimetric tests that use paper as a property-evaluation platform in combination with chemical reactions.

## 1. Introduction

Currently, the development of detection technologies for food has gained importance due to the necessity of evaluating its suitability for human consumption. The standards are established in the Codex Alimentarius, which indicates the levels of contaminants or adulterants allowed [1]. Likewise, indicators for food, energy, and water (FEW) have been considered to determine sustainability in the supply of affordable, reliable, and sufficient resources related to geographical demands [2]. For this reason, the design of sustainable devices has been considered in order to incorporate such technology in the food production chain to reduce the environmental impact caused by food production [3]. Diverse food products are part of the basic basket, such as milk, which is broadly consumed around the world [4]. Milk has been considered as a complete food because of its nutritional content, which is beneficial to human health [5]. Substances within milk such as protein, fat, and lactose are considered a major source of dietary energy [6]. Therefore, diverse methods used to determine quality and parameters associated with milk have been proposed, considering the complexity of the samples, pre-treatment, and milk adulterants [7].

Milk parameter evaluation has been broadly used; researchers have studied organoleptic characteristics like taste, smell, and raw material quality to evaluate milk. To achieve a measurable registration, several measuring instruments with different working principles such as optics, electrical and microfluidic techniques have been applied. In optics, spectrometry has been widely used to determine milk attributes such as fat content [8]. Likewise, it has been implemented in the determination of milk freshness by the identification of molecules causing milk aging [9]. The effects of thermal treatment have also been evaluated with spectrometry to identify differences between UHT (Ultra High Temperature) milk and raw milk [10]. These methods require specialized equipment, and the sample requires a specific treatment, which in many cases is not accessible for countries with limited technological resources. In the same way, electrical devices have been traditionally used for a long time to determine milk properties. Electronic tongues and noses have been considered in food spoilage detection in combination with chemometric tools [11]. In another example, impedance sensors have been used in the detection of milk adulterants in samples with different fat content [12]. However, these instruments require trained personnel and the cost associated with fabrication and materials represents a drawback for these devices. The size of detection devices has also gained importance to increase the portability of sensing equipment. As a consequence, different techniques have been developed using the ASSURED (Affordable, Sensitive, Specific, User-friendly, Rapid and robust, Equipment-free, Deliverable to all end-users) approach, which leads to device miniaturization [13]. In order to fulfill this requirement, there have been experiments in microfluidic device design, through ElectroChemical (EC), ChemiLuminescence (CL), and Fluorescence techniques [14,15,16,17], which are used in biomarker detection. Although these methods have detection accuracy, and meet portability characteristics, a drawback is the use of a specialized chemical substance, and the fact that final residuals are not easily deposed. Microfluidic systems have also been employed to analyze milk contaminants; however, a special chemical functionalization of the sample is required to separate fat, proteins, and other components [18]. Low-cost development and in situ response of these devices are required because milk preservation requires supervision to determine its suitability for human consumption. Furthermore, sustainability has been considered too, as part of the proposals established by the EGD (European Green Deal) in order to implement environmentally-friendly food systems [19]. Hence, a tendency of using paper as a sensing material has emerged due to its miniaturization capabilities and sustainability properties; therefore, researchers have used paper as a platform to develop microfluidic devices [20].

Microfluidic paper-based devices have been widely studied to reduce the size of the elements within the detection system. Characteristics such as portability and low cost have attracted the attention of researchers when developing analytical devices in a variety of applications [21,22]. Different techniques have been employed to evaluate properties in diverse substances using colorimetric, electrochemical, fluorescence, and chemiluminescence detection methods, which have sensed the contaminants contained in food and water [23]. For instance, microfluidic devices have been used to determine the presence of starch in milk through the addition of iodine in a wax-limited area, where a visible change in color is observed on paper substrate, and is measured employing image processing [24]. Furthermore, using paper as a bioactive platform has been studied in the detection of hydrogen peroxide, which serves to adulterate milk composition [25]. A drawback associated with such devices is the fabrication process, because it requires chemical substances in specific conditions and the use of wax barriers to limit the detection area. In addition, the design POC (Point-Of-Care) platform has to take into consideration the liquid composition, the analyte concentration, and the required accuracy and precision [26]. Another limitation associated with this kind platform is the pre-treatment of a complex sample, which can include component separation. Likewise, the authors in [27] reviewed diverse ways to measure different parameters in bioanalysis, such as blood, urine, etc., using spectra-based microfluidic devices that employ optical and electrical methods. They also highlighted the most common readouts such as pixel intensity, electrical current, fluorometry, etc. Nevertheless, the mechanical response studied through displacement measurement has not been addressed in that summary. The addition of electrodes in the paper surface has been studied in electrochemical sensing, where properties such as porosity, thickness, and material type have to be considered in the fabrication process [28]. Paper has also been employed in cantilever beam configurations in combination with other techniques, such as ELISA (Enzyme-Linked ImmunoSorbent assay), where a paper valve is activated through thermal response to supply fluid in a controlled manner [29]. However, paper is only used as a switch to activate or deactivate the liquid supply.

In this work, the electromechanical response of a paper-based cantilever beam is proposed to pinpoint different milk trademarks using liquid droplets with a volume of 20 μL to evaluate the sample bending response. The movement is induced by hygroexpansion due to the liquid transport among paper fiber. Flexural displacement is measured by image processing, and the area under the beam is analyzed and compared for three liquid samples. Likewise, the electrical response obtained is reported for each sample, where a change in voltage signal is observed when a droplet is deposited. Such response influences the paper mechanical response due to the heat induced by constant current supply. According to the obtained results, through statistical analysis applied to maximum displacement, it is possible to point out differences in milk trademarks as well as in the liquid samples used to compare the electromechanical response of the samples when interacting with these liquids. Additionally, the novelty of this work relies on the fact that it is possible to construct a device at a low cost, which provides information throughout the electro-mechanical response and it does not require the use of chemical reactions or specialized equipment.

## 2. Materials and Methods

In this section, a description of the materials used in this work is introduced. Afterward, the proposed methodology is described in five stages, which include the fabrication process, electromechanical response, measurement process, data processing, and analysis.

### 2.1. Materials

Filter paper from Triton Electronics Ltd., Dunmow, Essex, UK is used in the fabrication of the cantilever beam samples. Likewise, silver ink provided by INKcu is tapped for drawing the conductive path on the top surface of the beam. A blue laser is employed to cut the filter paper, and the image capture is performed using a digital microscope. Finally, a microcontroller from STMicroelectronics is employed to acquire voltage and current signals in the conductive path. The main properties of the materials used in this work are summarized in Table 1.

### 2.2. Methodology

**Figure 1:**
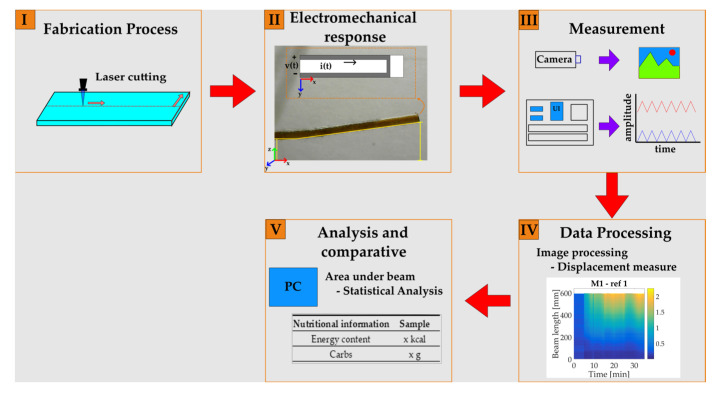
Schematic diagram for the proposed methodology. (**I**) Fabrication process; (**II**) Electromechanical response; (**III**) Measurement; (**IV**) Data processing; (**V**) Analysis and comparative.

A five-stage schematic diagram shows the proposed methodology of this work in Figure 1. The first step consists of the cantilever beam fabrication process (I), then the electromechanical response is evaluated through activations, which are induced by droplet deposition that triggers an upward displacement. Furthermore, a fixed current injection to the conductive path is connected (II). The third stage refers to the measurement performed through image capture and voltage readings (III), followed by the fourth stage, which corresponds to data processing (IV). Finally, the results are analyzed and compared with nutritional information (V).

#### 2.2.1. Fabrication Process

**Figure 2:**
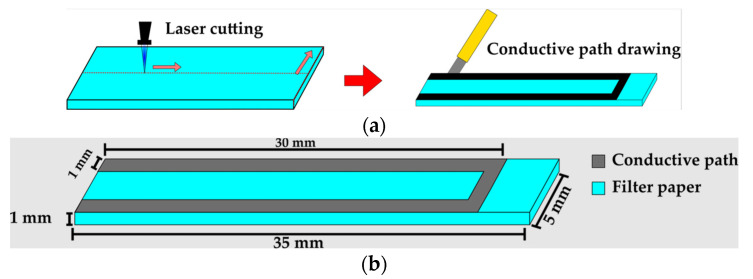
Fabrication process (**a**) Laser trajectory and hand-printed conductive path; (**b**) Paper-based cantilever beam final geometry.

The paper-based cantilever beam geometry is cut with a blue laser, to create a rectangular prism geometry with 5 mm of width, 35 mm of height, and 1 mm of thickness. The laser configuration is implemented using G-code to establish the trajectory coordinates as well as the velocity and laser intensity. A silver ink conductive path is added on the top surface using the INKcu silver ink that is manually painted over this surface (Figure 2a). The conductive path has 1 mm of width and 30 mm of length, the final geometry of the sample is shown in Figure 2b.

#### 2.2.2. Sample Selection

Three whole milk trademarks are selected to compare the electromechanical response when they interact with the paper samples. The samples are labeled as M1, M2, and M3. Nutritional information provided by each brand is presented in Table 2, which lists the most relevant parameters to identify the differences between each trademark. A 100 mL portion is selected to take five droplet samples of 20 μL from a sealed package. Likewise, three different liquid samples are listed, which correspond to commercial juice (S1), almond milk (S2), and distilled water (S3). Such samples are selected in order to compare the behavior of milk against other solutions, which have a significant change in nutritional information. Three experiments (E1, E2, E3) have been conducted per solution.

#### 2.2.3. Electromechanical Response

In this stage, the paper sample response is observed by adding microdroplets to it. The fixed end is placed at the start of the conductive path, where a constant current of 330 mA is delivered for 35 min in order to evaluate the behavior caused by four different solutions using micro doses of the liquid. As a hygroexpansion is produced due to liquid transport in paper fibers, an upward displacement is induced, which is captured by the acquisition system. Likewise, a potential difference is measured in the conductive path. The current flowing through the conductive path produces heat that impacts the evaporation time of the solution in the paper sample.

#### 2.2.4. Measurement

**Figure 3:**
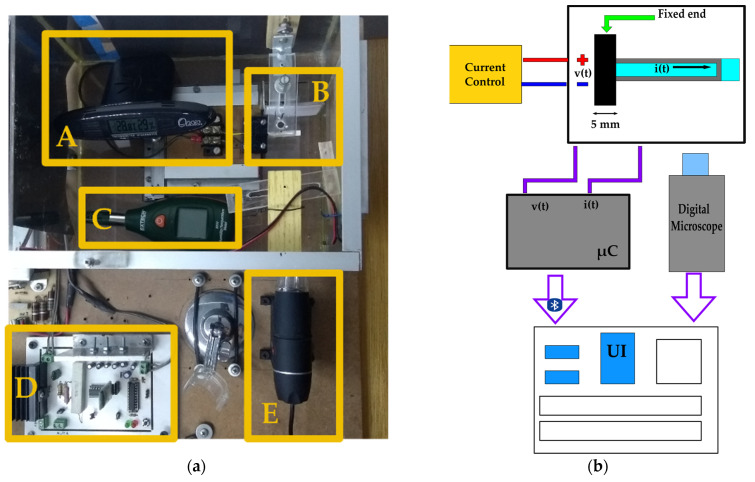
Closed environment system: (**a**) Main components of controlled environment: (A) Humidifier; (B) Beam Holder; (C) Hygrometer; (D) Current Control; (E) Digital Microscope; (**b**) Schematic diagram of acquisition system.

Experiments are developed in a closed environment; the main components are shown in Figure 3a. The system contains a humidifier (A) that allows establishing a Relative Humidity in a range between 30 and 45%RH (percentage of Relative Humidity), and a temperature ranging from 23 °C to 29 °C. Furthermore, the system contains a beam holder (B) that holds the fixed end of the sample and a Hygrometer (C) used to monitor temperature and humidity. A current control (D) was built to deliver a constant value of 330 mA across conductive path. Finally, a digital microscope (E) allows capturing beam profile, which is configured to take 8 shots per minute. The schematic diagram is shown in Figure 3b, firstly the current control is connected to the beam conductive path through the beam holder. Secondly, the measured data for *v(t)* (voltage) and *i(t*) (current) are set up using a μC (microcontroller), which sends the information via Bluetooth to a PC (Personal Computer). It should be highlighted that the current was measured to monitor its constant value. Hence, a UI (User Interface) is designed to set up the test time and to provide the front view of the cantilever beam profile provided by the digital microscope.

#### 2.2.5. Data Processing

**Figure 4:**
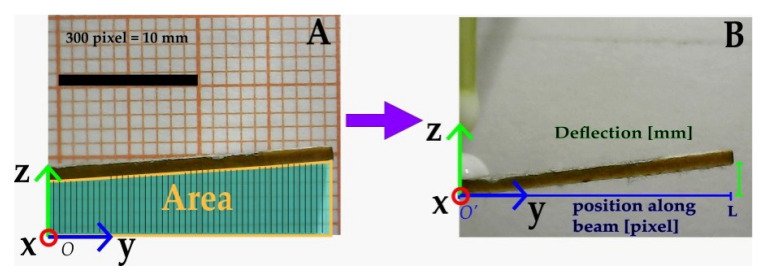
Image processing for deflection measurement. (**A**) Area under the beam; (**B**) Deflection measurement.

The acquisition system is set to acquire data for 35 min per experiment, a total of 281 images are processed using the software ImageJ where 300 pixels correspond to 10 mm; such a value was obtained using millimeter paper. Therefore, a macro is applied to measure the displacement from the image base initiating in *O* (origin coordinates) [0, 0] toward the beam underneath in the z-axis, as it is shown in Figure 4. A sweep along the image length in the y-axis (640 pixels) is achieved, the measurement data enclosures an area under the beam (A). Then data are processed to eliminate the offset, setting the origin in O’, and, thus, only the bending response is obtained by displacement measurement along the beam from O’ to L (B). Afterward, all the displacement data along the beam are plotted against time (minutes) and beam length (pixel).

**Figure 5:**
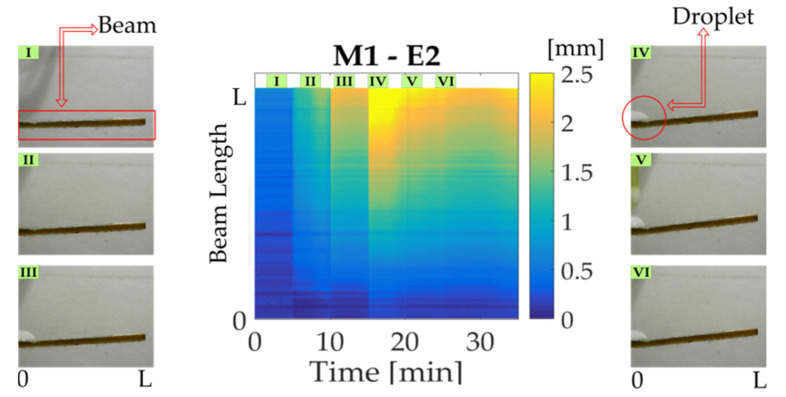
Colormap stated to activations induced by droplet deposition. (I) Start-up; (II) First activation; (III) Second activation; (IV) Third activation; (V) Fourth activation; (VI) Fifth activation.

In order to observe deflection values of the cantilever beam, a surface graph is obtained by the use of MATLAB©, where the x-axis corresponds to time, y-axis to image points along the beam from *O*’ to L, and z-axis to bending displacement. All measures obtained under the beam are plotted, setting an x-y view to observe the beam bending changes over time in a colormap, as it can be seen in Figure 5. A color bar next to the graph indicates a displacement value from blue to yellow that is concerned with minimum and maximum displacements. At first instance, the beam position corresponding to the first five minutes is shown in section (I), where it remains connected without solution addition. Then a first activation is induced by a 20 μL droplet, which triggers a movement upward of the beam, showed in section (II). After that, the droplets are deposited every five minutes as it can be noticed in section III, IV, V, and VI related to second, third, fourth, and fifth activations, respectively.

## 3. Experimental Setup

**Figure 6:**
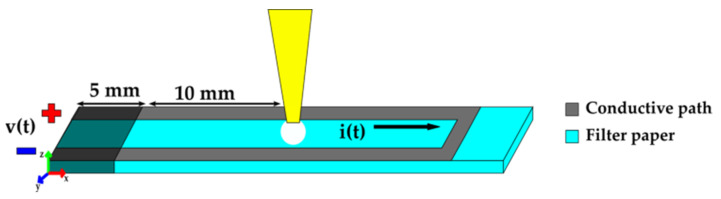
Experimental set-up for droplet deposition.

The procedure followed in this work to deposit sample solution is illustrated in Figure 6. The sample is inserted 5 mm in length within the beam holder. Hence, the current control is connected to the conductive path, and signal measurements (*v*(*t*), *i*(*t*)) are carried out. Each droplet is deposited into the cantilever beam, at a distance of 10 mm from the fixed end. Each test sample is connected for 35 min to a power supply that is set to deliver a constant DC (Direct Current) of 330 mA. The voltage and current across the conductive path are measured by the acquisition system, in order to observe signal response with the solution. At the start of each test, the cantilever beam sample remains connected for five minutes to evaporate moisture contained in the paper. After that, a droplet solution of 20 μL is added, then an activation occurs by the liquid transport in paper fibers, which provokes an upward displacement of the cantilever beam. The droplet deposition is performed every five minutes to produce five activations in the sample.

## 4. Results and Discussion

In this section, the results obtained by image analysis are introduced as a data matrix represented with the colormap, which indicates a displacement value, along the beam (y-axis) and time (x-axis). A color bar on the right side indicates a color degradation that goes from blue to yellow, corresponding to minimum and maximum displacement, respectively. The study case consists of three experiments (E1, E2, E3) performed with every milk trademark and liquid sample, in total six study cases are evaluated by measuring displacement. At first, milk response and liquid samples are described, according to the values obtained per experiment. Secondly, a statistical analysis is carried out for each sample type, computing the mean, median, mode, and the RMS (Root Mean Square) value.

### 4.1. Milk Trademark

**Figure 7:**
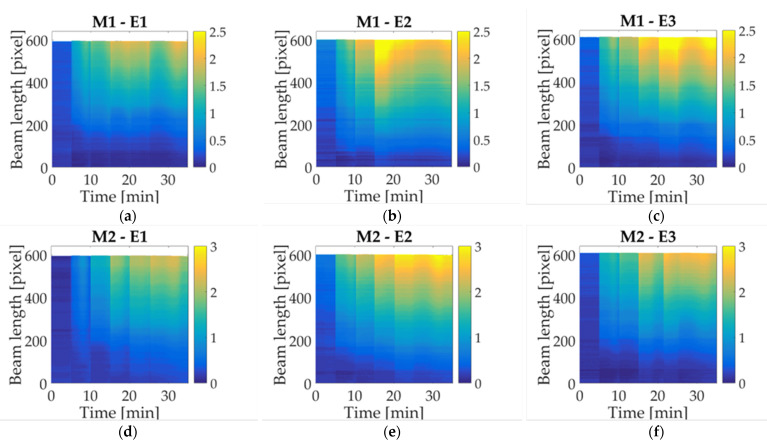

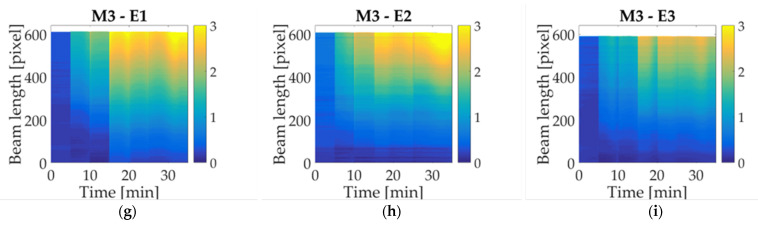
Colormap displacement along the beam for three milk trademarks: (**a**) M1 experiment 1; (**b**) M1 experiment 2; (**c**) M1 experiment 3; (**d**) M2 experiment 1; (**e**) M2 experiment 2; (**f**) M2 experiment 3; (**g**) M3 experiment 1; (**h**) M3 experiment 2; (**i**) M3 experiment 3.

The first case study corresponds to the M1 trademark, the results per experiment are shown in Figure 7a–c. As can be seen, during the experiment initial time, the beam remains in a start-up position (blue-colored), where the moisture contained in the paper is evaporated while connected to a constant current. After the first droplet deposition, a displacement increase can be observed in the cantilever free side. This behavior is repeated with every droplet until the fifth deposition, where it begins to decrease, until the final minutes, where a recovering up-ward can be noticed. The performance described is similar to the next milk study cases with a significant difference at the ending time. Figure 7d–f refer to the second milk trademark (M2); in this case, a change in maximum reached displacement is noted. Besides a gradual increment is observed with each droplet deposition, presenting a slight decrement after the fifth activation. However, the displacement goes up again in a short time. Likewise, the third study case (M3), indicated in Figure 7g–i, shows a displacement increase for every activation in a similar way to M1 and M2. It is worth mentioning that maximum displacement along the beam is concentrated at the cantilever free side. However, it is possible to notice a change between trademarks through statistical analysis.

Milk trademarks share a similar behavior regarding the upward displacement of each droplet deposition. As can be observed in Figure 7, the colormap associated with the maximum displacement is concentrated at the beam free-end, at the end of the experiment. Such performance can be inferred toward milk solution, where the change in reaching displacement is associated with the trademarks. That is explained with nutritional information shown in Table 2, where, despite that the samples are classified as whole milk, a variation of content is noticeable. The additives contained in milk study cases influenced the beam deflection; this behavior can be observed in the statistical analysis.

### 4.2. Liquid Sample

**Figure 8:**
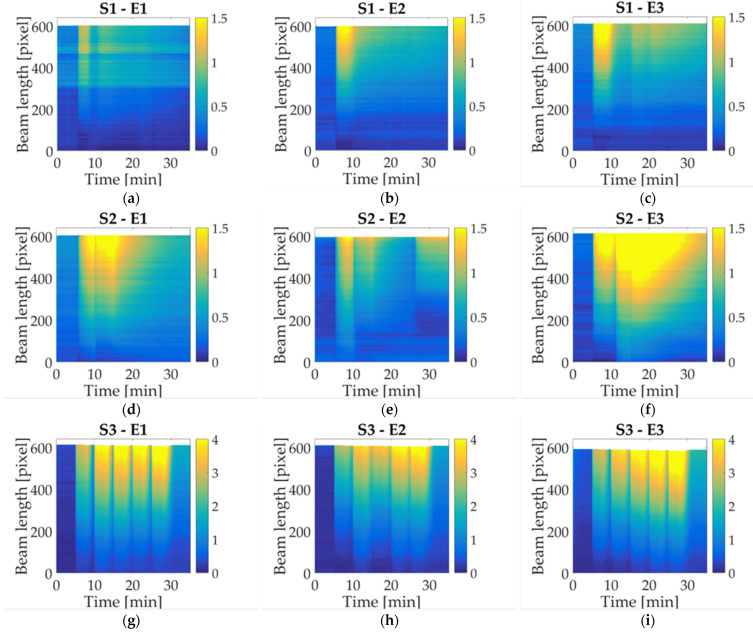
Colormap displacement along the beam for three liquid samples: (**a**) S1 experiment 1; (**b**) S1 experiment 2; (**c**) S1 experiment 3; (**d**) S2 experiment 1; (**e**) S2 experiment 2; (**f**) S2 experiment 3; (**g**) S3 experiment 1; (**h**) S3 experiment 2; (**i**) S3 experiment 3.

In these study cases, three different liquid samples are used to compare the response among milk trademarks. At first, the behavior of commercial juice (S1) is shown in Figure 8a–c, where an upward displacement is observed. When the first droplet is deposited, the sample reached a maximum value within five minutes. After that, it can be observed that, for the next depositions, the highest value is not reached again, and the deflection is reduced until the end of the experiment. Following with almond milk (S2), a similar response is observed as indicated in Figure 8d–f, but in this case, the beam remains for approximately 10 min in the maximum displacement after the first droplet deposition. Afterward, the deflection is reduced and oscillates between low and high values without reaching the upper limit again. An exception to the general almond milk behavior occurs in Figure 8f, where the highest value for 10 min can be seen. This could be explained as a consequence of the synchronization in the droplet application. Finally, a distilled water sample (S3) is evaluated through the same number of activations made in the previous experiment. An upward displacement is detected with each droplet deposition, reaching a maximum value, followed by a flexion reduction without going back to the reference level. This could be caused by the accumulation of water in paper fibers that end up swelling; therefore, water cannot be evaporated by the conductive path.

The liquid samples measured in this work showed a behavior completely different from responses caused by milk. In study case S1, a peak is reached at the beginning of droplet deposition, and then then the displacement goes down. An explanation for this could rely on the sugar content in commercial juice (S1), which is significantly higher than the one in the other solutions. Moreover, other properties may influence the mechanical properties of paper. Almond milk (S2) is analyzed as well to compare the response caused by the different liquids, both samples contain less energy than milk. However, there is a considerable difference regarding sugar content, where the juice has the highest value and almond milk has the lowest; such behavior can be seen in statistical analysis. The final study case corresponds to distilled water, which was selected as a reference liquid because it does not have additives. It should be noticed that the response obtained is not similar to the ones caused by the other liquids. As the sensing principle is based on a physical response, it is expected that physical features related to the fluid transport in paper have a strong influence on the bending response. In this case of beverages, these properties are density, viscosity, and surface tension.

### 4.3. Statistical Analysis

**Figure 9:**
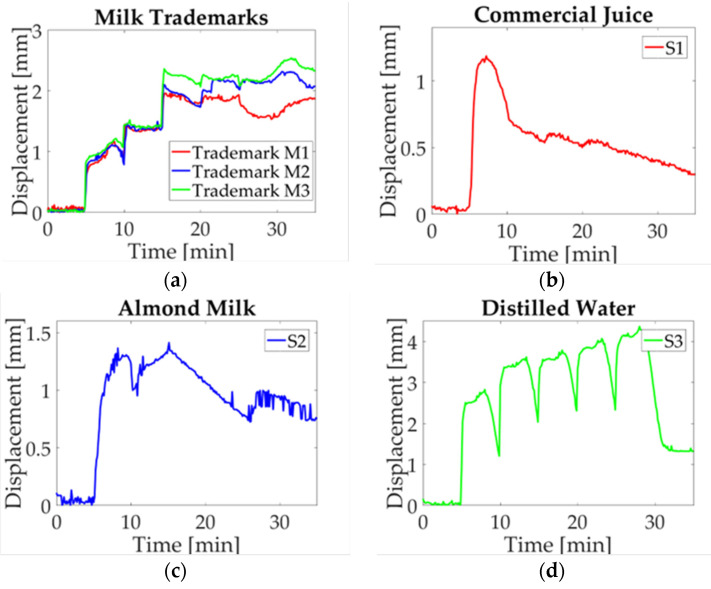
Mean value of statistical analysis of milk trademarks and liquid samples: (**a**) Milk trademarks; (**b**) Commercial juice (S1); (**c**) Almond milk (S2); (**d**) Distilled water (S3).

A study case description is presented in this section, the maximum displacement is acquired for each experiment. Three charts are obtained for each milk trademark and liquid sample. Then a single signal is obtained by averaging the three signals point-to-point in order to compare the electro-mechanical response. In Figure 9a, the obtained data for every milk trademark are shown. As it can be observed, they have a similar response during the first 15 min with a slight difference between them; this period corresponds to the start-up sample, the first and second droplet deposition. Subsequently, when the third droplet is deposited, a change in milk trademarks is visible. In the M1 study case, the displacement jumps to a maximum value, while M1 and M2 remain in a low displacement value until the fourth droplet is deposited. At this moment, a noticeable difference is observed, where M1 reaches a high displacement value and goes down for approximately five minutes, and then begins to displace upwards. For the M2 case, the displacement reaches its maximum value and oscillates around this value. Finally, in the M3 case, the value goes down and it goes upward recovered until the experiment is finished. It can be observed that the study case M3 stands out from other milk trademarks, whose results could be explained by energy content (57 kcal) and fat (2.8 g); these parameters have low values compared with other trademarks. Likewise, a significant difference in nutritional content for M1 and M2 is observed, such as energy content (61.4 kcal; 61 kcal) and carbs (5.6 g; 4.8 g), in which the value for M1 is higher than the value for M2.

Furthermore, the liquid samples are equally evaluated, in which a significant difference compared to milk performance is observed. The mean values computed for commercial juice (S1) are plotted in Figure 9b. Starting with a five-minute start-up, the 20 μL droplet is deposited, and displacement reaches the highest value. At this moment, the displacement begins to go down until it reaches the minimum displacement value. It should be noted that it is not visible in the graph when the next droplets are deposited. The next study case is almond milk (S2), represented in Figure 9c. In this case, it can be observed two peaks corresponding to the first and third activation. Afterward, a decrease can be seen until the last deposition where the displacement begins to soar without reaching the maximum value. In this plot, it can be seen that juice reached a maximum displacement of approximately 1 mm and almond milk reached a value of 1.5 mm. Therefore, it can be inferred that sugar content affects the beam deflection corresponding to 12.5 g for juice and 0.1 g for almond milk. Finally, a neutral solution is evaluated as a reference liquid selecting distilled water (S3), which is shown in Figure 9d. This sample is matched with all study cases. Here, a difference with respect to the maximum displacement is reached, and the return to the reference point is completely different from the one obtained in the other samples. Distilled water performance was explained in a previous work [30], where the return to the reference level is explained as a response to the conductive path addition, and water accumulation as a consequence of hygroexpansion in paper fibers.

**Figure 10:**
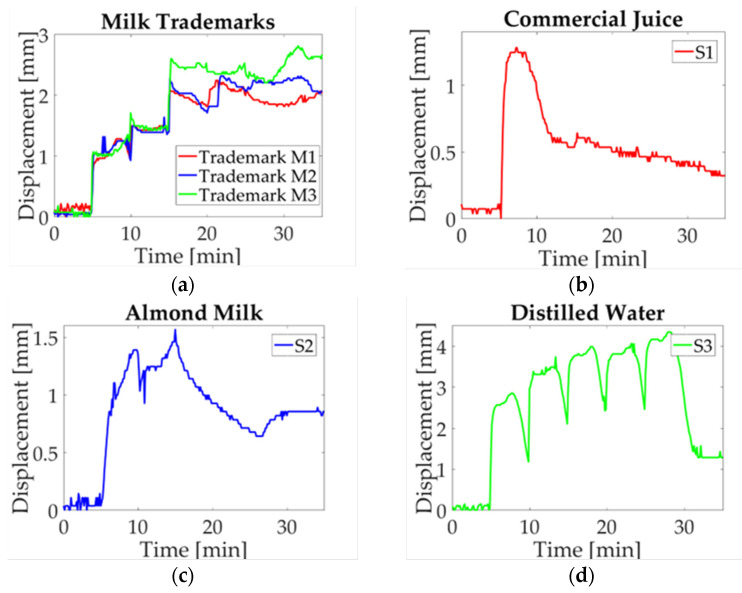
Median value of statistical analysis of milk trademarks and liquid samples: (**a**) Milk trademarks; (**b**) Commercial juice (S1); (**c**) Almond milk (S2); (**d**) Distilled water (S3).

In Figure 10a, milk median displacement is assessed; as it can be seen, a variation among trademarks is observed. In study case M3, the displacement measurement is sticking out above M1 and M2, in which a change is observed at the start of the fourth activation in the twentieth minute of the experiment. At this point, a separation occurs between M1 and M2, where the first goes down and the second goes up. The study case S1 is shown in Figure 10b, it can be observed that a peak is reached in first droplet deposition and then decreases to a minimum value. Likewise, almond milk (S2) and distilled water (S3), shown in Figure 10c,d, respectively, present a similar performance of that in Figure 9c,d. Based on the definition of median as the value that corresponds to a data middle value, which is not affected by extreme values, it can be concluded that there is not a significant change per solution sample; this is because the behavior observed in these plots is similar to the one of the mean values.

**Figure 11:**
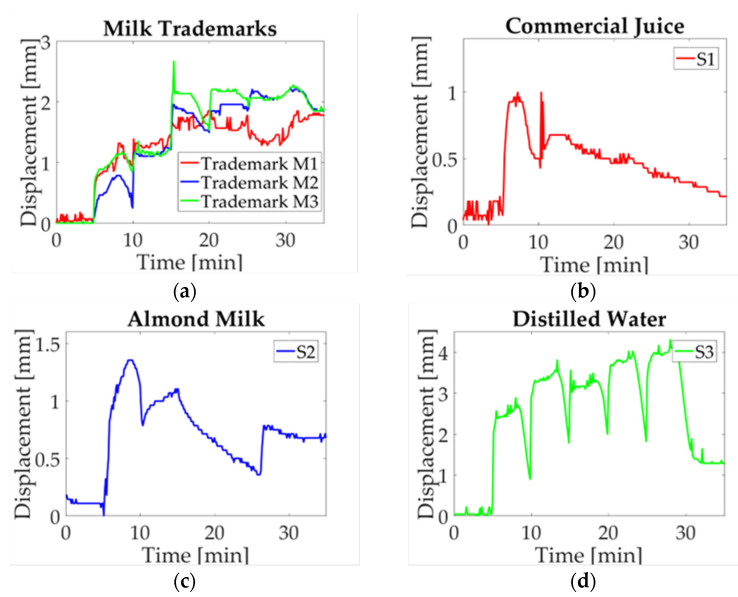
Mode value of statistical analysis of milk trademarks and liquid samples: (**a**) Milk trademarks; (**b**) Commercial juice (S1); (**c**) Almond milk (S2); (**d**) Distilled water (S3).

The mode graph shows a lot of variation along the results obtained for each solution sample, as can be seen in Figure 11. Despite that the mode is the most repetitive value of data distribution, in this case, a change in performance among analyzed signals is observed. Such behavior can be explained as a consequence of droplet deposition, which is manually added to the beam. Since a time-lag for activations is present, and only three values are used to calculate mode, the result observed in the plot is not similar to previous ones. In Figure 11a, the tendency shows a difference in milk trademarks; however, it is not observed for trademark M1 at the activation moment. Likewise, the study case S1 (Figure 11b) presents a variation due to the laser cut on paper; for this reason, there are no repeatable data because the measured displacement has an offset. For almond milk (S2), shown in Figure 11c, a similar circumstance is observed due to the last experiment where a time-lag in deposition occurs. Finally, distilled water (S3) is introduced regarding the displacement induced (Figure 11d), just like in previous cases, data are not repeatable though the behavior is the same.

**Figure 12:**
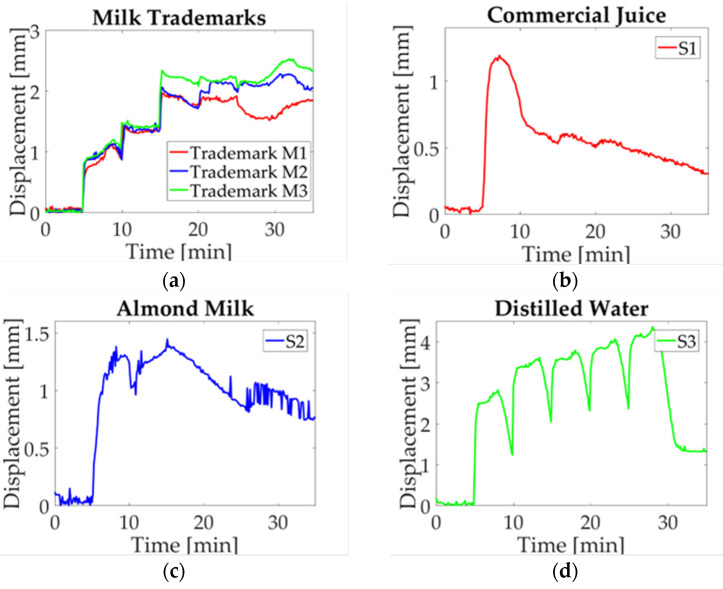
RMS value of statistical analysis of milk trademarks and liquid samples: (**a**) Milk trademarks; (**b**) Commercial juice (S1); (**c**) Almond milk (S2); (**d**) Distilled water (S3).

The last statistical value corresponds to RMS as it is observed in Figure 12. The parameter is defined as the absolute value of the square root of the mean squares [31]. In this case, a positive value can be seen in Figure 12a, where a difference between milk trademarks is visible. Similarly to what is observed in the mean plot, trademark M3 sticks out from the trademark M1 and M2. For liquid samples, it is possible to pinpoint the behavior associated with each solution sample. For example, Figure 12b shows the study case for commercial juice, which shows the same behavior as in the previous plot. Likewise, almond milk (Figure 12c) and distilled water (Figure 12d) have the same performance as the one shown in these plots. The statistical values selected to evaluate the paper response are commonly used to compare the behavior of repeated experiments, in order to approximate the behavior for each sample.

The results obtained from the deflection measurements and statistical analysis could be considered as a guideline to identify differences in milk trademarks. Firstly, the colormap shown in Figure 7 has a similar response for each sample regarding the upward displacement with every droplet deposition. The samples selected in this work are classified as whole milk; however, a difference in the maximum displacement mean is noted as can be seen in the graphs obtained by statistical analysis. The nutritional information provided by the manufacturer features a variation in the content for each trademark, which is reflected in graph behavior (Figure 9). A prospective of this work relies on the possibility of indicating differences not only in milk trademark with slightly variations in nutritional content, but also adulterations or decomposition of milk.

### 4.4. Electrical Response

**Figure 13:**
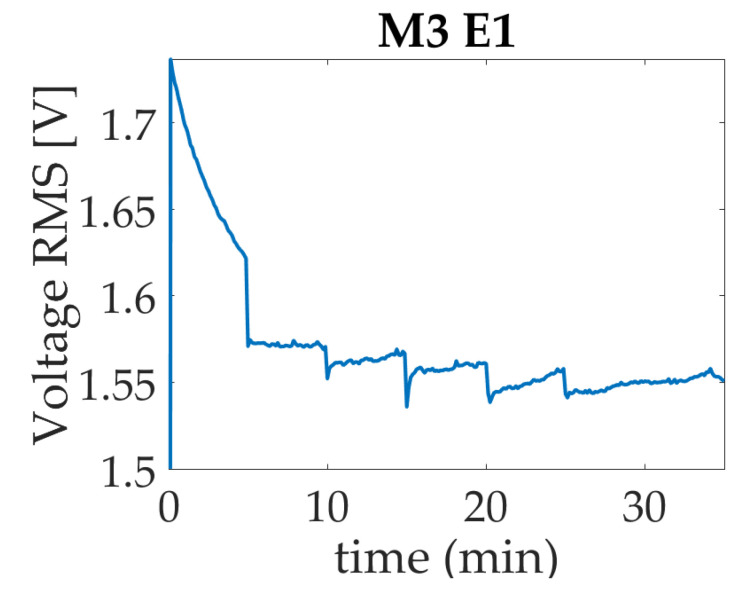
Voltage response for study case trademark M3.

The voltage RMS measure per experiment shows a similar performance in each one; for example, Figure 13 presents the results for the M3 case. In this graph, it can be observed that the signal begins with the five-minute start-up, then goes down until it is stable. At the moment that droplet is deposited in minute five, besides the voltage variations in the conductive path, the current remains steady. In the next activations, which occur in 10, 15, 20, and 25 min, a voltage decrement is observed due to ink conductivity when the droplet is deposited, which is bond with the micro-dose forming a parallel in the terminal. For this reason, the resistance is modified, as can be inferred from Figure 13 causing an increase in temperature that leads to the evaporation of the liquid sample.

## 5. Conclusions

According to the results obtained in the colormap matrix, the value measured in the area under the beam is concentrated in a specific zone, which allows identifying milk samples, beverages, and distilled water. Additionally, a statistical analysis was performed in order to compare the electro-mechanical response in the paper-based cantilever beam. The parameters evaluated, such as mean and RMS, showed a behavior that is associated with each solution. In these plots, a significant change in reaching a high value can be observed as well as the behavior over time through the activations. In the same way, the median and mode computed show variations; however, the results can be associated with the study cases. Furthermore, it is noted that in the milk trademarks, distinctions in the graphs are obtained despite their classification as whole milk. In conclusion, this working principle can be used to pinpoint differences between beverages and milk samples. It could be highlighted that this system represents a novelty due to the low-cost fabrication process, which can be potentially used to indicate the solution type in future works as milk tester.

## Figures and Tables

**Table 1:** Main properties of materials used in this work.

Material	Properties
Paper filter	Width: 70 mm
	Height: 90 mm
	Thickness: 1 mm
Silver Ink	Silver content: 60%
	Density: 1.2 g/mL
	Sheet resistance: 0.01 Ω/sq
Camera	Image sensor: CMOS
	Resolution: 640 × 480 pixels
	Focus range: 15 mm–40 mm
Laser	Power: 500 W to 12 V
	Color: Blue
Color: Blue	Component: MSP430G2553
	ADC: 10 bits resolution
	UART: 230,400 bps
	Memory: 512 Byte RAM and 16 KB Flash
	Clock: 16 MHz

**Table 2:** Nutritional information.

Nutritional Information	M1	M2	M3	S1	S2	S3
Energy content	61.4 kcal	61 kcal	57 kcal	50 kcal	19.6 kcal	NA *
Carbs	5.6 g	4.8 g	4.8 g	12.5 g	0.6 g	NA *
Sugar	5 g	4.8 g	4.8 g	12.5 g	0.1 g	NA *
Protein	3 g	3.1 g	3.1 g	0 g	0.7 g	NA *
Fat	3 g	3.3 g	2.8 g	0 g	1.6 g	NA *
Saturated fat	1.8 g	2.1 g	1.8 g	0 g	0.1 g	NA *

* NA: Not Applicable.

## Data Availability

The data presented in this study are available on request from the corresponding author.
